# Pleiotropic Effects of GLP-1 and Analogs on Cell Signaling, Metabolism, and Function

**DOI:** 10.3389/fendo.2018.00672

**Published:** 2018-11-23

**Authors:** Jordan Rowlands, Julian Heng, Philip Newsholme, Rodrigo Carlessi

**Affiliations:** School of Pharmacy and Biomedical Sciences, Curtin Health Innovation Research Institute, Perth, WA, Australia

**Keywords:** GLP-1, signaling, diabetes, metabolism, cell function and integrity

## Abstract

The incretin hormone Glucagon-Like Peptide-1 (GLP-1) is best known for its “incretin effect” in restoring glucose homeostasis in diabetics, however, it is now apparent that it has a broader range of physiological effects in the body. Both *in vitro* and *in vivo* studies have demonstrated that GLP-1 mimetics alleviate endoplasmic reticulum stress, regulate autophagy, promote metabolic reprogramming, stimulate anti-inflammatory signaling, alter gene expression, and influence neuroprotective pathways. A substantial body of evidence has accumulated with respect to how GLP-1 and its analogs act to restore and maintain normal cellular functions. These findings have prompted several clinical trials which have reported GLP-1 analogs improve cardiac function, restore lung function and reduce mortality in patients with obstructive lung disease, influence blood pressure and lipid storage, and even prevent synaptic loss and neurodegeneration. Mechanistically, GLP-1 elicits its effects via acute elevation in cAMP levels, and subsequent protein kinase(s) activation, pathways well-defined in pancreatic β-cells which stimulate insulin secretion in conjunction with elevated Ca^2+^ and ATP. More recently, new studies have shed light on additional downstream pathways stimulated by chronic GLP-1 exposure, findings which have direct relevance to our understanding of the potential therapeutic effects of longer lasting analogs recently developed for clinical use. In this review, we provide a comprehensive description of the diverse roles for GLP-1 across multiple tissues, describe downstream pathways stimulated by acute and chronic exposure, and discuss novel pleiotropic applications of GLP-1 mimetics in the treatment of human disease.

## Introduction

While its gene was first cloned in 1983, and protein product approved as a therapeutic agent for Type 2 diabetes mellitus (T2D) in 2005, the mammalian glucagon-like peptide-1 (GLP-1), its modes of action, and various analogs, have been and are still widely studied. As it is a highly attractive T2D therapy, the major known functions of the incretin peptide GLP-1 and analogs are based on studies delineating its role in the endocrine pancreas. GLP-1 acts through binding to its receptor (GLP-1R), triggering a downstream signaling cascade able to induce a potent stimulation of glucose stimulated insulin secretion (GSIS) in β-cells, as well as inhibition of α-cell glucagon release. GLP-1 analogs, such as Liraglutide and Exendin-4, unlike endogenously produced GLP-1, are not rapidly degraded by Dipeptidyl peptidase-4 (DPP-4) and, therefore, can induce sustained therapeutic actions, that otherwise would not be possible due to the exceedingly short half-life of endogenous GLP-1 in circulation. GLP-1R is a B class G-protein-coupled receptor abundantly expressed in the pancreas and central nervous system, but also detected in lower levels in the gut, kidneys, lungs, liver, heart, muscle, peripheral nervous system, and other tissues (1). Upon binding to the receptor, GLP-1 and its analogs also initiate a variety of additional anti-diabetic effects, including, but not limited to, reduction in gastric emptying, increase in satiety and inhibition of food motivated behavior, replenishment of insulin stores, as well as cytoprotective and anti-inflammatory actions on β-cells (2–9). To initiate these beneficial effects, however, GLP-1 must first be secreted from either the enteroendrocine L-cells, the preproglucagon (PPG) neurons located in the nucleus of the solitary tract of the brain stem, or, as reported recently, the α-cells in the pancreas (10–14). Upon ligand binding, GLP-1R initiates a cascade that involves activation of membrane bound Adenyl Cyclase (AC) and consequent production of cyclic adenosine monophosphate (cAMP). Downstream of cAMP formation, several signal transduction pathways can be initiated, which generally require activation of either one or both of the cellular cAMP effectors, Protein kinase A (PKA) and exchange protein directly activated by cAMP (EPAC) [reviewed in (15, 16)].

GLP-1R mediated effects arise as a consequence of the immediate signaling cascade, which can impact insulin secretion and calcium flux in a rapid post translational modifications based manner (4, 17), and/or, the late stage or chronic effects, which can operate through modulation of gene expression and cellular metabolism (18–21). To date, the vast majority of studies have tended to focus on the acute impact of GLP-1R activation. More recently, research has begun elucidating the consequences of chronic GLP-1R stimulation (19, 22–25). Long-lasting GLP-1 analog treatments are now in regular clinical use, and their impact, safety and efficacy are well-established and extensively reviewed (25–31). However, a succinctly summarized and current understanding of the signaling mechanisms and metabolic impact of chronic GLP-1R agonist activity on β-cells, and more broadly, across other tissues is both essential and lacking. To this end, our review outlines the current knowledge in regards to GLP-1R activation, subsequent signaling events, and discuss recent findings, firstly with respect to the well-characterized pancreatic β-cell, followed by effects on other cell and tissue types.

## Acute effects of GLP-1 in β-cells

Glucose enters the pancreatic β-cells via the transporter, *Glucose transporter 2* (GLUT2), moving down a concentration gradient from the capillaries. In the cytosol glucose is phosphorylated by the enzymes glucokinase/hexokinase (glucokinase is the predominant isoform in the β-cell), after which it enters the glycolytic pathway. Rapid catabolism of glucose via glycolysis and mitochondrial TCA cycle activity generates ATP (32, 33). The subsequent increase in ATP/ADP ratio leads to a closure of ATP-sensitive K^+^ channels, intracellular accumulation of K^+^ ions and subsequent membrane depolarization, causing an influx of Ca^2+^ via voltage dependent Ca^2+^ channels (VDCC). This Ca^2+^ influx, along with elevated ATP, results in exocytosis of the plasma membrane docked immediate release pool (IRP) of insulin granules, a sub-pool of the readily releasable pool (RRP) which contains ~1–5% of available insulin granules (16, 34). This is the main driver behind β-cell 1^st^ phase stimulus-secretion coupling, since it is the products of glucose catabolism that ultimately drive insulin exocytosis. This release is rapid, and is known to peak at around 10 min from the initial glucose challenge, whilst the second phase of insulin release, which is sustained, consists in the release of granules from the larger Reserve pool (RP), containing ~95–99% of insulin granules, and lasts until glucose stimulation ends (30–60 min under normal physiologic conditions) (16, 35). Before the trafficking and release of the RP granules occur, granule competency must be achieved, and this is believed to occur through granule acidification resultant from an increase of H^+^ and Cl^−^ ions and processing of pro-insulin into mature, releasable, insulin (36).

**Figure 1:**
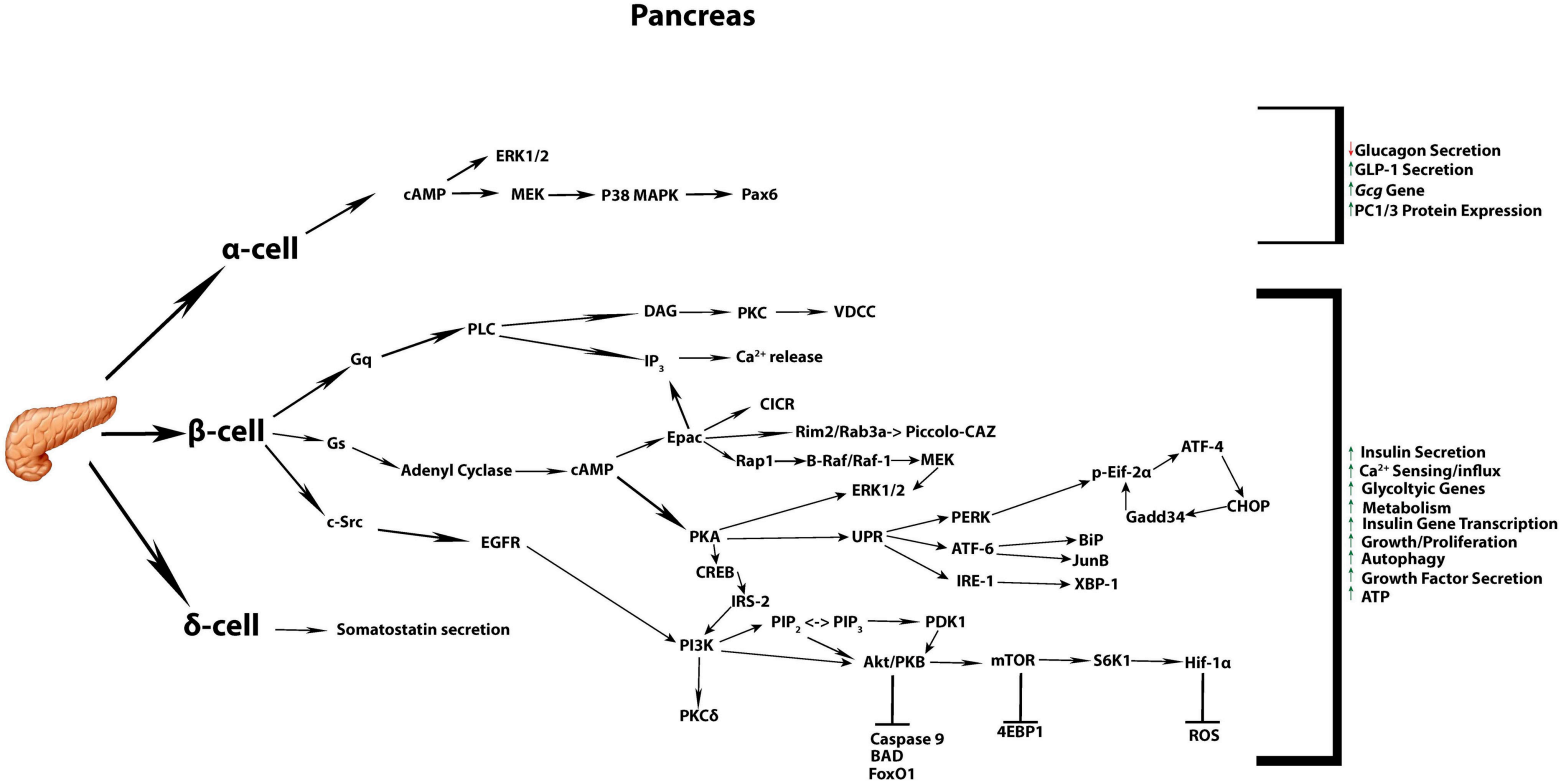
Commonly accepted GLP-1 signaling in the pancreatic tissue. Summary of the most commonly known signaling cascades activated by GLP-1 in the three different endocrine cell types α, β, δ, and their overall impact on diverse cellular processes.

In pancreatic β-cells, GLP-1R stimulated pathways act promptly (seconds to minutes) to potentiate glucose-dependent insulin release. This is achieved by a rapid increase in cAMP, which is accompanied by direct activation of PKA and EPAC. These two effectors of cAMP signaling modify several targets within the secretory machinery, with the net effect to synergistically enhance the amount of insulin secreted in response to glucose stimulation (15, 16). Indeed, several independent mechanisms are also reported to act in concert in order to result in enhanced insulin secretion, as discussed below (Figure 1).

Activation of PKA by cAMP results in release of its two catalytic subunits from the two anchoring regulatory subunits from specific cellular locations and anchoring proteins. Activated PKA can directly phosphorylate the sulphonylurea receptor (SUR1 as well as a regulatory subunit of K^+^ATP channels, thereby reducing SUR1 affinity to ADP, and increasing activity of K_ir6.2_, respectively (37). This, in turn, leads to channel closure and increased accumulation of intracellular K^+^ ions (9), influx of Ca^2+^ and promotion of insulin secretion in response to GLP-1 stimulation.

Another cAMP effector, EPAC, is implicated in K^+^ATP channel regulation. Kang, et al. demonstrated that activation of EPAC reduces the concentration of ATP required to achieve closure of K^+^ ATP channels (38). This indicates that in the presence of active EPAC, lower concentrations of ATP promote membrane depolarization and subsequent insulin granule exocytosis. Indeed, acute exposure to EPAC can stimulate insulin granule exocytosis and maturation, through sensitization of the ryanodine receptors and activation of the calcium sensing complex (16, 39, 40). EPAC aids insulin priming and release via facilitating formation of a Rim2/Rab3a complex via Rim2/EPAC interaction (3, 41–43). Rim2/Rab3a complex interacts with the Ca^2+^ sensor Piccolo-CAZ (cytoskeletal matrix protein that associates with the active zone) to facilitate vesicle exocytosis at the cytoplasmic surface of the insulin granule (3, 41, 42, 44). However, enhanced vesicle mobilization, priming, and subsequent exocytosis is not only regulated by the EPAC pathway, but also directly by PKA. PKA can facilitate insulin secretion through regulation of Ca^2+^ secretion, whereby PKA sensitizes the inositol triphosphate receptor leading to release of Ca^2+^ from intracellular stores (16, 45–47). PKA has also been reported to accelerate the competency and mobilization of vesicles from the reserve pool of insulin to the readily releasable pool and, thus, enhance Ca^2+^-dependent exocytosis in mouse pancreatic islets (16, 34, 48, 49). Indeed, a recent report described that PKA activity is required for glutamate uptake into the insulin granules, with glutamate uptake potentiating insulin release (50). Cytoplasmic glutamate can be derived via the malate-aspartate shuttle following pyruvate mitochondrial metabolism. Since glucose metabolism is absolutely required for GLP-1-induced stimulation of insulin secretion, the latter mechanism represents a clear link between glucose metabolism and GLP-1 action via PKA to amplify insulin secretion.

## Chronic effects of GLP-1 in β-cells

It is perhaps unsurprising that GLP-1 therapeutics show greater efficacy compared with traditional diabetic medicines, due to their potential to address not only acute stimulation of insulin secretion in response to a rise in blood glucose, but also slow the progressive loss of β-cell function and tissue mass in T2D. The beneficial effects of GLP-1 are resultant from cAMP mediated signaling, and ultimately activation of pro-survival cAMP responsive element binding (CREB) signaling, as well as the non-receptor tyrosine kinase/c-Src, transactivation of EGFR (5, 40, 51). CREB and EGFR pathways induce pro-survival and anti-apoptotic responses (52–57), including increased expression of anti-apoptotic genes (58), attenuation of ER stress (59), prevention of oxidative stress and fatty acid mediated toxicity (60). cAMP binds to PKA regulatory subunits, releasing and activating PKA catalytic subunits that cause phosphorylation of CREB at Ser^133^, promoting its activation and subsequent binding to genes containing palindromic CRE repeat sequences. Activated CREB regulates the expression of several genes essential for normal β-cell function, including the insulin gene (61). Additionally, EPAC can also exert late stage effects through the Rap1 protein, a small GTPase that regulates B-Raf/Raf-1 activation via a combination of residue phosphorylation (Ser^338^) and dephosphorylation (Ser^259^), which enables Raf to phosphorylate the mitogen-activated protein kinase (MEK). MEK, in turn, phosphorylates the threonine and tyrosine residues of the extracellular-signal regulated kinases (ERK) 1 and 2, which regulate gene expression, growth and differentiation (62, 63). In addition to protection against apoptosis, it has been proposed that GLP-1 induces β-cell proliferation in rodent cell lines and in isolated rodent islet cells (54, 55, 64). However, these findings have varying results in human cells, with a recent study identifying an age-dependent Exendin-4 induced signaling mechanism regulating β-cell proliferation (65). This study revealed that unlike adult human islets, juvenile human islets transplanted into an immunocompromised strain of mice suitable for xenograft studies retained their insulin secreting properties, and possessed a mitogenic response to a pharmacologically relevant infusion of Exendin-4. The mitogenic effect of chronic exposure of Exendin-4 in these transplanted mice was observed to arise from stimulation of the calcineurin/nuclear factor of activated T cells (NFAT) signaling pathway, leading to a variety of target genes essential for proliferation (65). Furthermore, this ability to enhance β-cell mass has been recently challenged in a study conducted in normoglycaemic mice where β-cell mass was decreased following 6 weeks of treatment with Liraglutide (66, 67). Therefore, GLP-1 analogs can have contrasting effects on β-cell proliferation depending on physiological context.

A physiological consequence of T2DM, due to peripheral insulin resistance, is high demand for enhanced insulin protein synthesis in the β-cell. It has been observed that sustained exposure to high insulin synthesis requirement results in ER stress due to protein overload and misfolding (68–72). The unfolded protein response (UPR) is the biochemical program initiated within the cell to counteract the accumulation of unfolded proteins in the ER lumen resulting in destabilization of ER homeostasis (73). The UPR initiates signaling cascades involving the luminal domains of three major ER resident proteins; Inositol requiring enzyme 1 (IRE1), protein kinase R (PKR)-like endoplasmic reticulum kinase (PERK), and activating transcription factor 6 (ATF6) (71, 74). The pathways so activated via signaling hubs attempt to re-establish ER homeostasis through transcriptional activation of genes involved in protein folding and protein degradation, as well as temporary attenuation of mRNA translation (75–77). This is achieved through downstream signaling events as, phosphorylation of eukaryotic translation initiation factor 2 alpha (eIF2α), resulting in global protein synthesis blockade, and alternative splicing of X-box binding protein 1 (XBP-1), a transcription factor involved in misfolded protein retrotranslocation and degradation (78–80). Failure to alleviate ER stress, and consequent prolonged UPR activation, leads to apoptotic cell death primarily through upregulation of the pro-apoptotic transcriptional factor C/EBP homologous protein (CHOP) (71, 81, 82). Several physiological and environmental insults associated with T2DM have been shown to induce ER stress in β-cells, these include hyperglycemia, dyslipidemia, inflammation and oxidative stress (83).

GLP-1 has been reported to alleviate glucotoxicity, lipotoxicity, excess nitric oxide (NO), Ca^2+^ depletion, oxidative stress, and cytokine-induced ER stress in both primary β-cells and cell lines through several downstream signaling mechanisms (59, 74, 84–86). For example, Yusta, B et al., demonstrated that GLP-1R signaling facilitates the shift from translational repression to translational recovery phase in a PKA-dependent manner (74). The recovery phase is concomitant with enhanced activation of ATF-4, CHOP and over-stimulation of *Gadd34* gene signaling cascade, which leads to eIF2α dephosphorylation. Furthermore, GLP-1 treatment leads to upregulation of spliced XBP-1 (sXBP-1), which is involved, along with ATF6, in enhancing ER function through activation of genes encoding molecular chaperones and ER-associated protein degradation (60–70, 74). In addition, GLP-1 protection from lipotoxic stress has been demonstrated to occur downstream from induction of the ER chaperone Binding immunoglobulin Protein (BiP) and anti-apoptotic protein JunB (86). Animal studies have recapitulated these findings, whereby diabetic mice treated with GLP-1 analogs displayed a significant reduction in biochemical markers of ER stress, increased expression of antioxidant genes and improved metabolic parameters (60, 74).

Most recently, GLP-1 has also been implicated in the regulation of autophagy in β-cells (87, 88). Autophagy, a mechanism that can promote cell survival during nutrient depletion, may also occur under basal and excessive nutrient conditions. This cellular process is characterized by the formation of autophagosomes, which can capture cytosolic components and fuse with lysosomes to promote the recycling and/or degradation of its contents. The process can be separated into four stages, initiation, nucleation, elongation, and fusion/degradation [reviewed in (89)]. Initiation is controlled by the mammalian Target of rapamycin (mTOR)/ AMP-activated protein kinase (AMPK)/ Uncoordinated (Unc)-51-like kinase 1 or 2 (ULK-1/2) axis, a crucial regulatory step leading to the activation of class III phosphatidylinositol 3-kinase complex, formation of the phagophore (a double membrane vesicle that encloses and isolates the cytoplasmic components during autophagy), and ultimately, recruitment of key proteins involved in the nucleation phase (90). Once the phagophore is formed, the elongation phase is undertaken where the phagophore captures the desired cargo for degradation. This is regulated by two ubiquitin-like reactions that act in concert to mediate the localization of key proteins to the developing autophagosome, and expansion of its membrane (91–93). Finally the autophagosome fuses with lysosomes, after which lysosomal enzymes initiate content degradation and nutrient and metabolite recycling (92). Autophagy provides essential components for energy production and biosynthesis during nutrient depletion. However, it also acts in a similar fashion by recycling of damaged organelles, unwanted proteins and foreign matter when adequate nutrients are available (88). In an environment with excessive nutrients, however, autophagy acts to remove unfolded proteins and toxic aggregates, thus facilitating ER homeostasis. GLP-1 can facilitate autophagy under chronic exposure to excess nutrients, whereby it prevents autophagosomal-lysosomal fusion impairment (87, 88). Similarly, Exendin-4 was reported to enhance lysosomal function, consequently leading to improved autophagosome clearance in a rat model of tacrolimus-induced diabetes whereby autophagosome accumulation causes islet injury (94). In the latter study, *in vivo* Exendin-4 treatment decreased tacrolimus-induced hyperglycemia, oxidative stress, and apoptosis. In parallel, it was demonstrated that β-cells from treated animals presented with reduced autophagosome numbers and decreased autophagy related protein expression. Thus, GLP-1R signaling could be interpreted as inhibiting autophagy, however, it most likely depicts its positive effects on autophagosomal-lysosomal fusion and, therefore, as a positive mediator of autophagic flux. It should be noted, however, that GLP-1 induced changes can vary depending on the underling mechanism of stress. For example, while usually promoting autophagy, treatment with GLP-1 analogs in a high fructose fed rat model resulted in apparent inhibition of β-cell autophagy, and increase in β-cell mass and function (95). The underlying mechanisms and downstream molecular mediators through which GLP-1 influences autophagy remain to be better characterized.

Recently, several studies have significantly improved our understanding of the regulation of β-cell energy metabolism by chronic GLP-1R activation. Notably, acute vs. chronic effects of receptor activation and downstream cAMP signaling leads to two distinct waves of gene expression regulation in primary islet cells. The initial wave of gene expression occurs as rapidly as 2 h after cAMP elevation upon acute receptor activation, and is mediated by CREB. Sixteen hours from the initial stimulation, a second wave of gene expression regulation takes place, and is orchestrated by *Hypoxia-inducible factor 1* (HIF-1), a transcriptional factor that targets genes involved with glucose uptake and glycolysis (21). Van de Velde et al. reported that chronic GLP-1R activation led to metabolic reprogramming marked by increased ATP production and upregulation of glycolytic enzymes, occurring as a result of late activation of subunit alpha of HIF-1 (HIF-1α) downstream of mTOR. This finding is consistent with a recent study demonstrating that depletion of HIF-1α or inhibition of mTOR impaired the effects of GLP-1R signaling on glycolysis (18). Whilst it is known that the Phosphoinositide 3-kinase (PI3K) /AKT (Protein kinase B) /mTOR axis is intimately linked to metabolic functions such as protein synthesis, glucose uptake, ATP production, nutrient transport, autophagy and cellular growth (96, 97), recent publications have identified novel effects of chronic GLP-1R stimulation which impact these pathways. For instance, GLP-1R agonists have been reported to promote secretion of insulin like growth factor-2 (IGF-2), and induced expression of its receptor (IGF1-R), which once stimulated activate downstream cascades including the PI3K/AKT as well as the cellular growth and proliferative mitogen-activated protein kinase (MAPK) pathway (19, 20, 98). The biosynthesis, secretion and subsequent activation of the IGF-2/IGF-1R autocrine loop is significantly enhanced by the presence of glutamine, and has been reported to protect β-cells against apoptosis, and increases β-cell glucose competence (20, 98, 99). However, whilst this autocrine loop stimulates PI3K/AKT activity and contribute to some of the pro-survival abilities of GLP-1 in β-cells, it does not seem to mediate the enhanced metabolic phenotype induced by chronic exposure to GLP-1R agonism. Rowlands et al. demonstrated that neither functional inactivation of IGF-2 nor silencing of its receptor by siRNA could mitigate the observed metabolic adaptations enacted by prolonged exposure to Exendin-4 (19). Thus, as the mitochondria and ER form structural and functional networks, the ability of GLP-1 to enhance metabolism may reduce ER stress by enhancing mitochondrial derived ATP and Ca^2+^ for utilization in the maintenance of ER homeostasis (46, 100, 101). Through elevation of cytosolic and intra-mitochondrial Ca^2+^, and mobilization of intracellular Ca^2+^, GLP-1 may mitigate the required Ca^2+^ transfer from ER to mitochondria, thereby sustaining mechanisms which facilitate protein folding (102–104). Therefore, we hypothesize that metabolic reprogramming in β-cells underlies the protective effects of GLP-1 under various stress conditions. In this scenario, enhanced metabolism can provide additional energy required to facilitate stress response and pro-survival mechanisms utilized by β-cells during challenging physiological conditions.

Although primarily studied in the pancreatic β-cell, the beneficial effects of GLP-1 and its analogs have recently been shown to be advantageous to a variety of tissues in several disease pathologies, such as the heart, liver, lung, muscle, and brain, as detailed below in the following sections. It remains to be clarified, however, as to whether the metabolic and pro-survival responses arising from chronic GLP-1R mediated signaling cascades are relevant to extra-pancreatic tissues as well.

## GLP-1 action in other tissues

### Skeletal muscle

**Figure 2:**
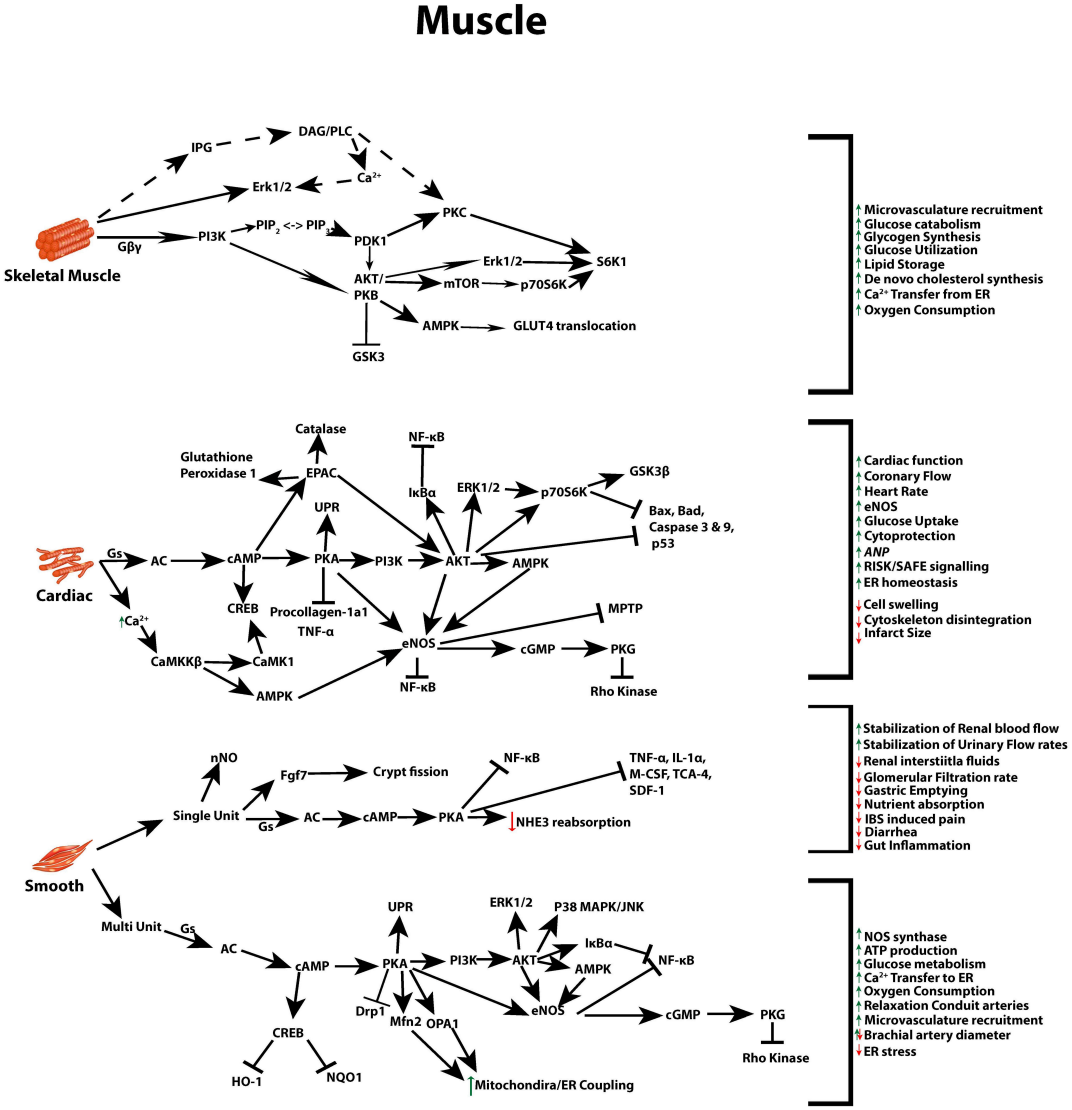
GLP-1 signaling across different muscle types. Multiple signal transduction pathways and their net impact arising from GLP-1R stimulation in skeletal, cardiac, and both multi and single unit smooth muscle.

Beneficial actions of GLP-1 differ between, skeletal, smooth and cardiac muscle, and again between the two subsets of smooth muscle, single and multi-unit cells (gastrointestinal/urogenital and vasculature cells, respectively) (105). Due to its ability to act on numerous pathways that can regulate glycaemia, weight, lipid metabolism, and blood pressure (Figure 2) (1, 29), as further outlined below, GLP-1R agonists, have been implicated and implemented as a potential therapy to address the increasingly prevalent pathologies associated with metabolic syndrome.

Studies assessing GLP-1's extra-pancreatic effects, such as its insulin-like actions, revealed that exposure of skeletal muscle to GLP-1R agonists enhanced glycogen synthesis, glycogen synthase α (GSα) activity, glucose metabolism and inhibited glycogen phosphorylase α activity in diabetic and non-diabetic rodent models, and human tissue (106–109). Interestingly, compelling evidence suggests that such muscle effects are independent of cAMP signaling. This was observed in rat and human muscle cells, but also in studies conducted in hepatocytes and adipocytes, potentially utilizing inositol phosphoglycans (IPGs) as the intracellular second messenger (108, 110, 111). While the amino acid sequence, same as that of the pancreatic GLP-1R, has been identified in multiple tissues (112), these studies identified that GLP-1 acted through a unique receptor distinct from that of the β-cell, which allowed this deviation from the canonical signaling of GLP-1. Whether this deviation from the non-canonical effects of the GLP-1R in muscle tissue are resultant from alternative splicing, the widespread hetero dimerization of B-family GPCRs, variations in ligand-receptor interactions or GLP-1 degradation products still requires further investigation (113–115).

To date, understanding of GLP-1 effects in skeletal muscle has mostly stemmed from the laboratory of Villanuevea-Penacarillo, who have revealed that GLP-1R agonists can induce PI3K/PKB (Akt), P44/P42 MAPK, p70S6K, and Protein kinase C (PKC) signaling pathways in skeletal muscle cells (116–119). Corroborating this findings, similar results were obtained in L6 myotubes and 3T3-adipocytes, that Exendin-4 promoted a PI3K dependent increase in insulin-stimulated glucose uptake (120). Other lines of evidence suggest that GLP-1R activation promotes skeletal muscle glucose transport independent of insulin through the AMPK signaling pathway and downstream activation of TBC1D1, a paralog of the phosphorylated Akt substrate AS160, thereby leading to translocation of GLUT4 to the plasma membrane (121–124). Akt is the canonical mediator of insulin-induced GLUT4 translocation. Although, it should be noted that the difference in signaling pathways from these latest studies may result from the extended duration of exposure to the GLP-1R agonist (122, 123). Interestingly, two of the above mentioned papers reported a rise in cAMP measured in muscle cells, as well as an increase in PKA contributing to a favorable metabolic phenotype in the studied muscle cells (121, 123). One possible explanation for these findings may be that, as in neurons and sperm, increases in cAMP could be a result of enhanced Ca^2+^ or even calcium/calmodulin-dependent protein kinase II activity which in turn can activate AC (125–128). While the ability for GLP-1 to impact skeletal muscle in regards to glucose catabolism and glycogen synthesis has been analyzed in depth, further mechanistic studies are required to fully elucidate the role for incretin hormones in regard to this tissue type.

### Smooth muscle and vascular tissue

Recent studies have described receptor dependent and independent effects of GLP-1 in smooth muscle, whereby exposure to physiological concentrations of acutely infused GLP-1 can relax conduit arteries in healthy humans, and recruit skeletal and cardiac muscle microvasculature. Dilatation of microvessels can facilitate insulin and nutrient delivery, tissue oxygenation, and glucose utilization (129, 130). This dilatory effect is believed to occur as a result of GLP-1 binding to the abundantly expressed, endothelial cell GLP-1R, triggering a downstream signaling cascade resulting in microvasculature recruitment via a NO-dependent mechanism (129, 131, 132). Additionally, the effect of the GLP-1 analog Liraglutide on endothelial cells has been evaluated in cultured human umbilical vein endothelial cells (HUVECS) to evaluate its impact on ER stress and apoptosis induced following overnight exposure to high glucose (133). The authors found that such treatment reduced apoptosis and ER stress through a mechanism which likely involves stimulation of the nuclear-encoded mitochondrial protein optic atrophy protein 1 (OPA1). It appears that the ability for GLP-1 to modulate mitochondrial metabolism is not limited to the β-cells. Indeed Morales et al. (134) reported that GLP-1 treatment stimulates mitochondrial activity in A7r6 vascular smooth muscle cells through recruitment of the ER to the mitochondria via the tethering protein Mitofusin-2 (Mfn-2). Enhanced Mfn-2 promotes ER-mitochondria co-localization and increases Ca^2+^ transfer from ER to the mitochondria, thus facilitating high demand for oxygen consumption and ATP production.

Studies conducted in rodent models have shown that stimulation of the GLP-1R led to changes in blood pressure (BP), depending on experimental model (29, 135, 136). In humans, one study reported an increase in BP over 2 h from healthy subjects following a single administration of GLP-1 (136), while another reported that chronic administration of GLP-1 analogs to patients with metabolic syndrome led to a reduction in BP [reviewed in (29, 137, 138)]. Such conflicting results may result from complex actions of GLP-1 on vascular smooth muscle and cardiac tissues in combination with its effects in the autonomic nervous system. Altogether GLP-1's multi-tissue actions can mediate alterations in BP, vasodilation and constriction, body weight, and heart rate (120). Nevertheless, the short- and long-term effects of GLP-1 on vascular smooth muscle are not completely understood and thus require further examination to ensure GLP-1 therapies can be utilized to their utmost potential.

Another mechanism by which GLP-1 therapies have been utilized to attenuate or partially attenuate metabolic syndrome is through their impact on diet and satiety (29, 130, 133, 139, 140). Notably, GLP-1's action on gastric emptying has been indicated to be enacted by reduced contraction in human intestinal muscles, and it occurs as a direct result of activation GLP-1R in the gastrointestinal (GI) tract (140), rather than for its ability to mitigate food motivated behavior through receptor activation in the hypothalamus or the hindbrain (141). However, the effects on gastric emptying appear to be short acting, since clinical studies and animal models of chronic administration of GLP-1R activators altogether suggest a negligible effect on long-term gastric emptying. Instead, evidence suggests that reduced weight gain occurs through direct actions on the pancreas, as well as through reduction of appetite mediated by central nervous system responses (141–143). Reducing the rate of gastric emptying, however, does not just impact satiety, but also delays the rate of entry of nutrients into the small intestine and their subsequent absorption, which therefore influence postprandial glucose metabolism, hormonal responses, and ultimately enhances GLP-1's anti-diabetogenic effects (144, 145). Slowing of small bowel motility was reported to occur in a GLP-1R and nitric oxide (NO) dependent manner, independently of both somatostatin and insulin, in fasting but not fed rats (146). Such effects have been reported in healthy (147), obese (148), diabetic (149), and critically ill human subjects (150). While gastric relaxation and postprandial gastric accommodation were reported to be mediated by vagal cholinergic pathways (151–153). Work by Amato et al. (154) validated these findings by demonstrating that acute administration of GLP-1 activated the GLP-1R in human colon cells and resulted in an inhibitory effect on large intestine motility through release of neural NO.

### Kidneys

A broad array of renoprotective properties have been reported from GLP-1 therapies. Positive effects in the renal tissue were observed both in diabetic and non-diabetic models of chronic kidney disease (CKD), as well as acute kidney injury (AKI) (155–160). Although not often considered as part of the metabolic syndrome, accumulating evidence has begun unearthing a link between the increasing morbidity and mortality rate in patients with kidney disease and metabolic syndrome (156, 159, 161, 162). This entwinement of kidney disease and metabolic syndrome complicates investigations with GLP-1 therapies due to the indirect benefits GLP-1R agonist therapies have on other tissues including but not limited to alterations in BP, glucose homeostasis, weight loss and insulin levels [reviewed in (155, 156, 161, 163)]. Adding to this scenario is the lack of agreement regarding the exact locality of GLP-1R expression in the kidney (1, 164), although it is generally accepted that in humans and rodents the GLP-1R is expressed in the renal vasculature and afferent arterioles, with some studies reporting receptor expression in the proximal tube and glomerular capillary, but not in the distal tubules (155, 165–168). It is evident from both clinical and animal studies that GLP-1 based therapies are beneficial to kidney function through increases in renal blood flow (RBF), urinary flow rate, prevention of rises in plasma creatinine, reduced tubular necrosis, an increase in renal interstitial fluids and glomerular filtration rate (GFR), as well as cytoprotective and anti-inflammatory actions (160, 161, 164, 166, 169, 170).

GLP-1R agonists are believed to cause these effects in the kidney through both direct kidney based GLP-1R activation, and indirect receptor actions, potentially through interactions with the nervous system (170), the renin angiotensin system (RAS) (155, 171–173), and regulation of atrial natriuretic peptide (ANP), a blood pressure and electrolyte regulator (173). Regardless of this lack of consensus in terms of indirect kidney responses to GLP-1 therapies, the direct actions of GLP-1R activation in the renal tissue are consistent. Acute exposure increases the diuretic and natriuretic excretion rate, which is in part dependent on inhibition of NaHCO_3_ reabsorption via a cAMP/PKA modulation of NHE3 (renal cortical Na^+^/H^+^ exchanger isotope 3) (166, 169). Furthering this, GLP-1's renal hemodynamic actions have been observed to alter GFR, potentially to regulate the filtered electrolyte load and volume (159, 174). In this sense, Exenatide acutely increased GFR and suppressed proximal tubular reabsorption in Wistar rats, resulting in approximate doubled early distal flow rate (175). Altogether these finding imply that Exenatide works as a diuretic at the kidney level. Similar results have been recently reported in humans (176).

Activation of the GLP-1R/cAMP/PKA pathway is also crucial in renal protection, with studies in a range of rodent models reporting a reduction in renal inflammation, renal fibrosis, and decrease in renal oxidative stress arising from the toxic milieu induced in metabolic syndrome (158, 161, 166, 177, 178). These pro-survival abilities are believed to arise from enhanced GLP-1 signaling leading to a reduced expression of the pro apoptotic markers caspase-3, and Bax/Bcl-2 (158), as well as reducing oxidative stress through increased expression of the oxidative defense gene heme oxygenease-1 (HO-1) (160, 178), and inhibition of NAD(P)H oxidase in a cAMP/PKA dependent manner (166). Activation of the GLP-1R signaling pathway has also been reported to reduce macrophage infiltration, potentially alleviating the associated increase in ROS and inflammation, as well as attenuating the progression of renal fibrosis through downregulation of ERK1/2 and its upstream activator transforming growth factor-beta 1 (TGF-β1) (160, 177). Despite these results, further studies are still required to fully elucidate the molecular mechanisms that mediate the reported attenuation in apoptotic and inflammatory pathways, particularly as accumulating clinical evidence highlights the potential of GLP-1R agonist therapies in DKD, ultimately urging for deeper understanding of cellular actions of these analogs in the renal tissue [reviewed in (156, 157, 159, 162, 179)].

### Adipose tissue

Although GLP-1 based therapies primarily aid weight loss through satiety, their usefulness is further extended by multiple studies implicating GLP-1R agonists as regulators of adipogenesis. Studies have indicated that GLP-1 based therapies can potentially influence whole body energy metabolism through their regulation of adipocyte development, acceleration of plasma clearance of glucose and triacylglycerol derived fatty acids, improvement of insulin signaling and stimulation of brown adipose tissue (BAT) thermogenesis (31, 142, 180–184). The GLP-1R in adipocytes was reported to activate the AC/cAMP signaling pathway, regulating apoptosis and pre-adipocyte proliferation through various cell signaling cascades including ERK, PKC and AKT, as well-altering the expression of peroxisome proliferator-activated receptor gamma (PPARγ) and its target genes (142, 185). GLP-1 may also act through a brain-adipocyte axis to modulate lipid metabolism in BAT, as well as white adipose tissue (WAT). In various rodent models, administration of GLP-1R agonists induced BAT thermogenesis through increased uncoupling protein 1 (UCP1), mitochondrial respiratory chain element *Cox4i1 (*Cytochrome C Oxidase Subunit 4I1*)* and PGC1α, independent of nutrient intake, as well as altering the expression of transcription factors involved in *de novo* lipogenesis (123, 181, 186). Interestingly, GLP-1 has also been shown to activate Adipose-resident invariant natural killer T (iNKT) cells, triggering fibroblast growth factor 21 (FGF21), a major player in iNKT cell induced weight loss (187).

While still in its infancy, and convoluted by the various interconnected pathways, studies investigating the effects of GLP-1 therapies in the adipose tissue of patients with obesity show promise, with trials replicating *in vitro* studies, and indicating a potential long term benefit of GLP-1R agonists therapies also in this important tissue. Deeper studies into the underlying mechanisms are warranted in order to specifically identify direct actions of GLP-1 agonists in BAT and WAT physiology and lipid metabolism.

### Heart

Given that both T2D and obesity represent important risk factors for cardiovascular disease (CVD), there is emerging interest to establish the potential cardiovascular benefits of GLP-1R stimulation. Even though the positive effects of GLP-1 analog therapies on the metabolic conditions described above could theoretically improve CVD outcomes, mounting evidence points that GLP-1 can also influence the cardiac tissue through direct receptor mediated responses. Indeed, it is recognized that the classical response initiated by GLP-1R activation leads to facilitation of cardiac function through enhanced glucose uptake, improved coronary flow, and in mice, secretion of atrial natriuretic peptide (ANP), a blood pressure and electrolyte regulator (163, 173, 188). However, studies to define the mechanism through which GLP-1 directly influences cardiac tissue are complicated by its broad actions in other tissues, such as blood vessels. For instance, a study conducted by Mells et al. (189) indicated that liraglutide treatment was able to reverse BP increases and cardiac hypotrophy resulting from a high fat diet (HFD) induced obese mouse model. The study, however, did not dwell further into the underlying mechanisms, making it particularly difficult to distinguish between the direct effects of the treatment in the cardiac muscle from those emanating from other tissues. Recent data published by the Drucker Laboratory and colleagues have indicated that some of the contrasting results of GLP-1 on the cardiovascular system in regards to both increasing and decreasing heart rates, and BP, are partially mediated by neurological signaling (120). Remarkably, in studies where the GLP-1R was conditionally disrupted only in mice cardiomyocytes (GLP-1R^CM−/−^), pre-treatment with liraglutide could still promote cardioprotection, increased survival and reduced infarct size following ischemia-reperfusion injury, suggesting these outcomes are not mediated directly by cardiomyocyte GLP-1R activity (190). Glucagon-like peptide (GLP)-1 (9–36) amide-mediated cytoprotection in ischemic-perfused mice was blocked by the GLP-1R antagonist exendin-9–39 but did not require the known GLP-1 receptor (190–193). Thus, the direct and indirect mechanisms which underpin the beneficial effects of GLP-1R agonism on cardiac injury remain to be clarified.

In an effort to address these gaps in knowledge, multiple groups have endeavored to define the role of GLP-1, its analogs, and related peptides (11, 194), in protecting cardiomyocytes and endothelial cells from injury. Indeed, GLP-1R activation leads to the re-establishment of ER homoeostasis, cytoprotection, and restoration of signaling pathways disrupted by diverse stress stimuli (139, 191, 192, 195). For example, liraglutide treatment corrected the decreases in eNOS, the endothelial nitric oxide synthase, responsible for most of the vascular nitric oxide production in a HFD model of cardiac dysfunction in mice (195). This is of particular importance as NO is crucial in a pathway that regulates the synthesis of the ubiquitous intracellular second-messenger cyclic guanosine 3′,5′-monophosphate (cGMP). It has been reported that cGMP can activate two types of effector molecules in cardiovascular system, cGMP-dependent protein kinases (PKGs) and phosphodiesterases (PDE), which can stimulate cellular proliferation, mediate vaso-relaxation, and inhibit hypertrophy (196). Importantly, the effects in eNOS were accompanied by significant decreases in cardiac tissue TNF expression and NFκB activation (195). These effects were confirmed to be direct actions of the GLP-1R agonist in heart and vascular tissues since liraglutide also prevented palmitate-induced lipotoxicity in isolated mouse cardiomyocytes and primary human coronary smooth muscle cells *in vitro*. Together these data indicate that GLP-1R activation can activate multiple complementary protective and pro-survival mechanisms in cardiac cells, and endothelial cells. These findings are further supported by larger animal trials in which GLP-1 induced reduction in infarct size after ischemia-reperfusion (IR) injury (197), improved left ventricular function, and altered heart rate and BP when infused into dogs with pacing-induced cardiomyopathy (198). Furthermore, GLP-1 is hypothesized to activate ischemic conditioning (IC) through reperfusion injury survival kinase (RISK) and survivor-activating factor enhancement (SAFE) pathways (199, 200). Activation of this conditioning pathway post GLP-1R stimulation has been shown to reduce infarct size, improve cardiac function and enhance AKT activation and Bcl-2, an important anti-apoptotic protein, expression after IR injury in pigs (201). IC is interconnected with the mitochondrial K-ATP channel (mK-ATP) (202, 203) as well as the ATP derived metabolite adenosine, which activates the adenosine receptor and its signaling pathway leading to ischemic preconditioning (193, 204, 205). Although still unclear, the role of GLP-1R signaling cascades in the activation of conditioning pathways may include hijacking these subcellular pathways. Furthering this, the GLP-1 mediated relaxation of *ex vivo* rat aorta described by Green et al. (194), was lost upon K-ATP channel blockage, indicating a link between GLP-1R activation induced IC.

Initial human trials mimicked cellular and animal model studies, with GLP-1 therapies improving left ventricular (LV) function in patients with acute myocardial infraction (AMI) and serve systolic dysfunction (206). The promising results of this pilot study were followed in 2006 by an additional study demonstrating that chronic GLP-1 infusion over 5 weeks can improve LV function and quality of life in diabetic and non-diabetic participants (207). As these improvements were seen in both diabetic and non-diabetic groups, glycemic control in the GLP-1 treated group was deemed not to be a contributing factor to the beneficial effects. Since these early studies, treatment with GLP-1 analogs has been noted to improve hemodynamic recovery in patients undergoing coronary artery bypass grafting (208), protect against ischemic LV dysfunction (209), prevent hyperglycemia during cardiac surgery (210) and reduce reperfusion injury (211–213). Finally, data from recent large scale cardiovascular outcomes in T2DM trials revealed a significant reduction in cardiovascular death rates in GLP-1 analog treated patients (214, 215). Research into GLP-1 therapies on cardiac tissue continues to represent an expanding field, with the potential for a broad range of therapeutic applications beyond cardiovascular outcomes to be realized through an understanding of the underlying mechanism of action.

### Liver

**Figure 3:**
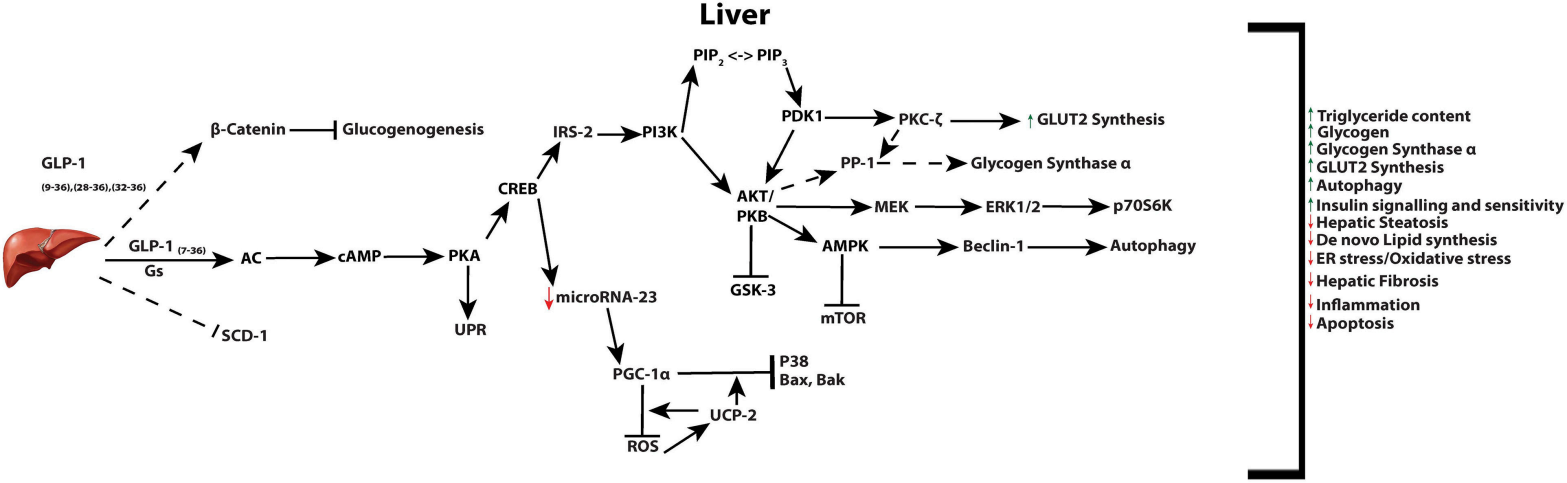
Active GLP-1 and its truncated products signaling in the liver. Active GLP-1 and its cAMP dependent pathways (solid arrows), as well as hypothesized (dashed arrows) truncated product induced signaling and their respective net effects.

Of the body's organs, levels of GLP-1 are recognized to be highest in the liver owing to transport of the incretin through the hepatic portal vein. The therapeutic effect of GLP-1 and its analogs on restoring hepatic function impaired by a variety of insults is supported by *in vivo* and *in vitro* studies (27, 216). Changes induced from GLP-1 or its analogs, in the liver, regulate a variety of processes including, hepatic gluconeogenesis, glycogen synthesis, and glycolysis (Figure 3) (1, 216, 217). In rodent models, GLP-1R agonist based therapies have been reported to increase both glycogen and glycogen synthetase α, through PI3K, PKC, PP-1 (type 1 protein phosphatase), pathways in isolated hepatocytes (218), as well as acting in an insulin-like manner to inhibit glucagon-induced glycogenolysis in perivenous hepatocytes (219). While the presence of the GLP-1R is still controversial in hepatocytes, GLP-1R expression at the protein level has indeed been reported in transformed human hepatocyte cell lines, HuH7 and Hep-G2, as well as primary human hepatocytes (220). However, regardless of the presence of the receptor in hepatocytes, direct receptor-ligand mediated actions in the liver remain controversial with some research groups proposing that observed benefits are a result of receptor independent events (221–225). Mechanisms may include GLP-1 degradation products GLP-1_9−36_, GLP-1_28−36_ or GLP-1_32−36_ which may be transported through the plasma membrane without the involvement of a receptor, and activate AC and Wnt signaling [reviewed in (226)].

To date, studies in animals and humans have provided evidence for the potential of Liraglutide to improve hyperlipidemia, liver fibrosis and inflammation, non-alcoholic fatty liver disease (NAFLD), as well as reduce liver fat content in T2DM patients (227–230). Acute exposure of Sprague Dawley (SD) rats to GLP-1R activators controlled hepatic glucose production (HGP) through a gut-brain-liver neuronal axis, discussed later, involving GLP-1R stimulated duodenal mucosal PKC-δ activation (231). In this context, Exendin-4 was found to inhibit key gluconeogenic enzymes and enhance hepatic insulin signaling. Exendin-4 was also reported to improve hepatic steatosis and insulin sensitivity in ob/ob mice, which was paralleled by reduction in oxidative stress and genes associated with fatty acid synthesis (232). Female APOE^*^3-Leiden.CETP mice, a model with human-like lipoprotein metabolism, were fed a cholesterol-containing diet and subsequently treated for 4 weeks with exendin-4. Utilizing a mouse model with human-like lipoprotein metabolism and western-type diet for 5 weeks to induce atherosclerosis, Exendin-4 treatment reduced inflammation within the liver and vessel well (233). Use of Exendin-4 in this mouse model limited the progression of hepatic inflammation and atherosclerosis through a reduction in macrophage influx and adhesion to the liver and vessel wall. These findings are further supported by a study revealing that GLP-1 analogs impact the production of triglyceride-rich lipoproteins in normoglycaemic men (234).

The hepatic actions of GLP-1 may be mediated through signal transduction of the AMPK/mTOR pathway. This was reported in a study showing that improvement of hepatocyte steatosis by liraglutide involves autophagy and its controlling AMPK/mTOR pathways (235). By inducing autophagy, GLP-1 therapies can relief the burden in the ER, reduce ER-stress, and subsequent hepatocyte apoptosis (236). Understanding the impact of GLP-1 treatments on the liver is crucial, as perturbations to both cellular lipid and very-low-density lipoprotein (VLDL) metabolism are associated with development of hepatic insulin resistance, obesity and diabetes. Recently, chronic stimulation of the GLP-1R led to increases in the mitochondrial uncoupling protein 2 (UCP2), an anti-mitochondrial oxidative stress gene, and the master mitochondrial biogenesis regulator and protective gene, peroxisome proliferator activated receptor-gamma coactivator 1α (PGC-1α) (237). Such gene expression changes were hypothesized to be mediated through downregulation of the microRNA-23 and result in improved hepatocyte survival through reduction in mitochondrial ROS production, inhibition of P38 activity, and decrease in expression of apoptotic genes Bak and Bax (237, 238). Combined with previous knowledge that PGC-1α and UPC2, play critical roles in mitochondrial metabolism (239), these data provide additional support for the hypothesis that the improved metabolism resultant from GLP-1R stimulation underlies the pro-survival abilities of GLP-1 signaling pathways.

### Brain

**Figure 4:**
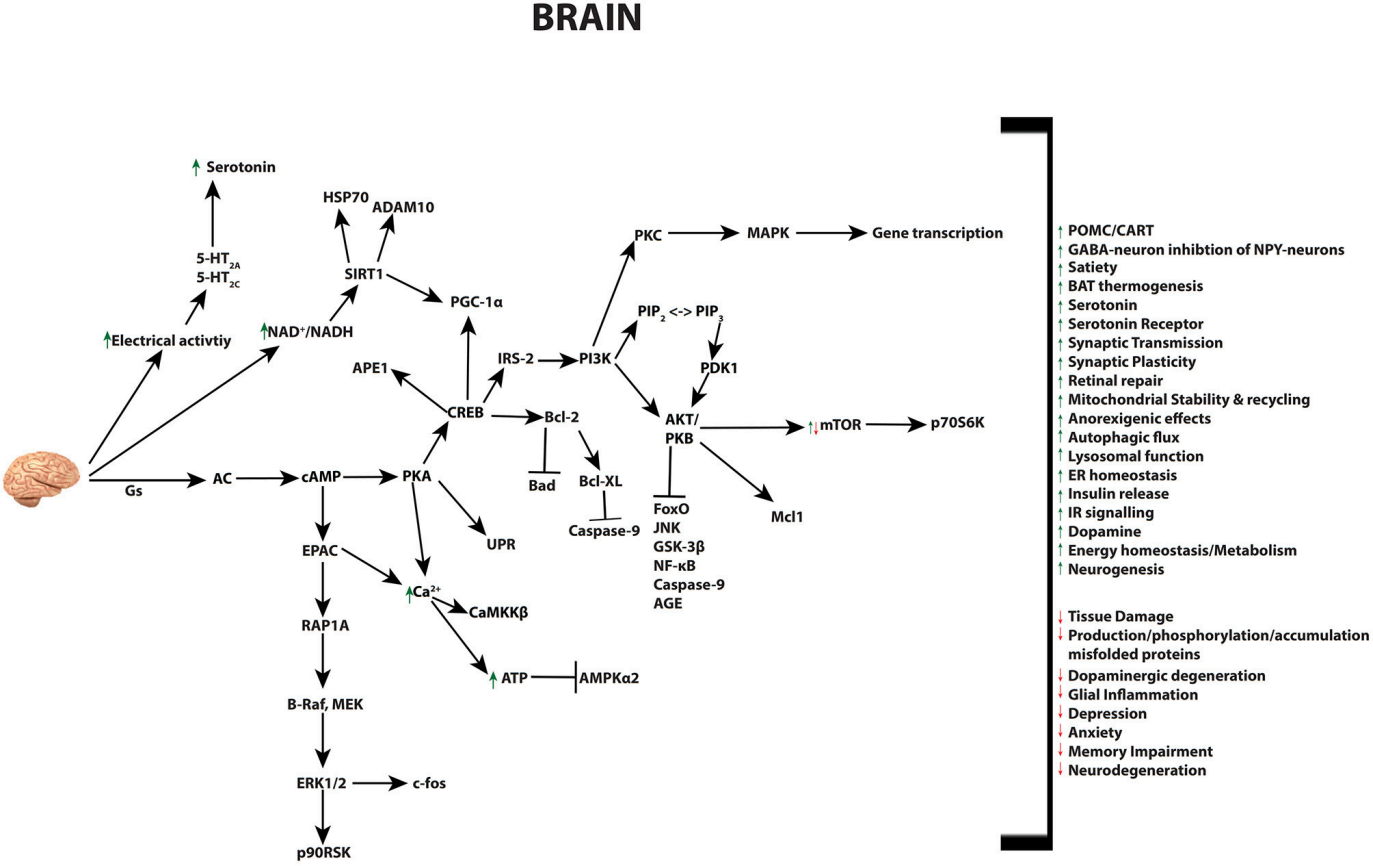
Pleiotropic effects of GLP-1 signaling in the brain. GLP-1 and its analogs activate diverse signaling pathways in the brain, leading to a plethora of neuroprotective outcomes.

GLP-1 and its receptor agonists are able to influence a variety of brain functions, including but not limited to: satiety, thermogenesis, blood pressure, neurogenesis, neurodegeneration, retinal repair, and altering energy homeostasis (Figure 4) (26, 30, 240–245). The GLP-1R is expressed in cells of the cerebral cortex, hypothalamus, hippocampus, thalamus, substantia nigra, circumventricular organ (CVO), cerebellum, and brainstem nucleus. This pattern of gene expression in the nervous system is evident in rodent, non-human primates, as well as humans (26, 241, 242, 244, 246–248). Studies with mice have identified the source of GLP-1 to derive from preproglucagon neurons of the nucleus of the solitary tract within the brainstem (249). These neurons project to the thalamus, hypothalamus and cortical regions, and induce the release of GLP-1 by various stimuli in a mechanism similar to L-cells of the small intestine (30, 31, 240, 243). Gut-derived GLP-1 can cross the blood brain barrier (BBB) and bind receptors in the circumventricular organs of the brainstem, however its short half-life is believed to limit its function within the brain. Instead, it most likely influence the brain indirectly, through vagal nerve fibers in the enteric area, whereby it transmits metabolic information to the nucleus of the solitary tract (NTS)—neurons responsible to control brain regions known to mediate feeding behavior (250). Recent research has revealed that GLP-1 analogs, due to their extended half-lives, can reach the BBB and have distinct effects to endogenous GLP-1 in the brain (26, 250). These effects include the well-investigated anorexigenic effects, outlined below, as well as a range of neuroprotective abilities that have led to the use of GLP-1R agonists as therapies in human trials for a range of neurodegenerative diseases as discussed in detail further below.

Studies in rats using intracerebroventicular (icv) administration of GLP-1 or its analog Exendin-4, alone or in combination with the receptor antagonist Exendin (9–39), have shown that activation of the GLP-1R inhibits food intake and weight gain; such effects are attributed to changes in brain controlled hormone secretion (251–254). Similar findings have been recapitulated in studies of obesity in humans, and have highlighted GLP-1 based therapies as potential anti-obesity treatments. The role for GLP-1 signaling in satiety is understood to be a consequence of GLP-1R signaling attenuating the release of the orexigenic neuropeptides Neuropeptide Y (NPY) and agouti-related peptide (AgRP), as well as promoting the anorexigenic neuropeptides pro-opiomelanocortin (POMC) and cocaine- and amphetamine-regulated transcript (CART) (250, 254–256). These neuropeptides are produced by arcuate nucleus of the hypothalamus (ARC), a critical regulator of energy balance, feeding behavior, and body weight (257, 258). GLP-1 is believed to alter food intake through this pathway, with an acute receptor induced modulation of AMPK activity in the hypothalamus. Secher et al. (259), supports these findings, but also discovered that while the GLP-1 analog Liraglutide directly stimulates POMC/CART neurons, it indirectly inhibits NPY/AgRP neurotransmission via GABA-dependent signaling. This liraglutide induced weight loss, altered food intake and conditioned taste aversion occurs through CNS receptors rather than the vagus nerve, area postrema, paraventricular nucleus, or visceral nerves (259, 260). Novel data has recently surfaced proposing that activation of astrocyte GLP-1Rs may play a role in energy balance in the CNS and GLP-1s anorectic effect, although the mechanics underlying this new finding still require deeper investigation (261). Furthermore, native GLP-1 infusions in the CNS, have been shown to modulate thermogenesis of BAT, via enhanced sympathetic nervous system (SNS) activity (183). Both chronic and acute CNS infused GLP-1 promoted BAT activation and subsequently glucose and triglyceride uptake, via activation of AMPK in the hypothalamic ventromedial nucleus in rodents (181, 182). GLP-1 is also able to upregulate alternatively activated (M2) macrophage-related molecules in human monocyte-derived macrophages (HMDM). This M2 macrophage activation enhanced production of anti-inflammatory factors was also found noted to enhance adiponectin secretion from adipocytes and derived from GLP-1 induced activation of the activator of transcription 3 (STAT3) which can further contribute to its protective abilities against metabolic syndrome (262). More recently, GLP-1 and its analogs have been shown to act in the dorsal raphe, whereby GLP-1R activation alters serotonin turnover and the 5-hydroxytryptamine 2A (5-HT_2A_) and 5-HT_2c_ serotonin receptors in rats (263). Dorsal Raphe GLP-1R stimulation induces hypophagia and increases the electrical activity of the serotonin neurons in this region, indicating that serotonin may be a new neural substrate for GLP-1 activity and aid GLP-1s ability to reduce appetite, and body weight through bioenergetics metabolism (263). Whilst the ability of GLP-1 and its analogs to stimulate serotonin receptors in humans has yet to be tested, various studies have employed neuroimaging techniques, such as functional magnetic resonance imaging (fMRI) to assess GLP-1 actions in human brain. These studies recapitulated animal studies, where infusion of GLP-1 analogs was found to; attenuate neuronal activity in reward processing areas, reduce appetite and hedonic feeding in healthy volunteers (264), as well as obese and T2DM patients (265, 266). Whilst weight loss as a result of GLP-1 therapies in humans is believed to be primarily resultant from their inhibitory effect on food intake; distinct neuronal responses should be taken into account when investigating the effects of GLP-1 in neurodegenerative diseases. Chronic inflammation of the brain is a known pathophysiological hallmark of various neurodegenerative diseases, including Alzheimer's disease (AD), multiple sclerosis (MS), and Parkinson's disease (PD), all demonstrated in animal models to benefit from GLP-1 mimetics therapy (14, 26, 30).

Mental illnesses and neurodegenerative diseases not only negatively impact on a patient's quality of life through their impairment of motor functions, they can enhance dementia and depression, which are often refractory to treatment (267). This resistance to treatment may be influenced by accumulating evidence that implicates a link between neural inflammation and the pathology of depression (268, 269). In addition, analysis of patients with psychiatric illnesses has revealed an alteration to crucial intracellular signaling pathways including the AKT/GSK-3 (glycogen synthase kinase) pathway (269, 270). This data along with observation that GLP-1R signaling enhanced levels of serotonin, dopamine (DA), and their receptors (263, 271), has potentiated the use of GLP-1R agonists as a management strategy for mental illness and neurodegenerative diseases. While initial *in vitro* studies have begun to reveal the mechanistic effects of GLP-1 in regards to neurodegenerative diseases, as outlined further below, both animal and human trials have already taken advantage of GLP-1's beneficial actions. Several animal studies have been undertaken with two studies showing that chronic treatment in rats is associated with reversal of depression-like behavior and acute treatment induced anxiety-like behavior (270, 272), while an alternate study indicated no GLP-1 induced changes in either behavior (273). Such contrasting results raise caution in the design and implementation of human trials which evaluate the therapeutic effects of GLP-1 and its analogies in the treatment of neurodegenerative diseases.

Although several recent studies have begun to elucidate the impact of GLP-1 analog therapies on the progress of neurodegenerative disorders, no human clinical trials have directly measured the impact of GLP-1 analog therapies on mental health disorders. Despite this, several trials using GLP-1 analogs for T2DM have a battery of neuropsychological tests as secondary outcome measures that may provide insights into GLP-1's impact on mental illness (274). Whilst many studies are still underway, only one recent clinical study analyzing the effect of the liraglutide analog in regards to Alzheimer's disease has been reported (NCT01469351) (275), and two clinical trials and one population based nested case-control study assessing Parkinson's Disease and GLP-1R activity (NCT01971242, NCT01174810) (28, 267, 276–278). Findings from the AD study in Denmark indicated that GLP-1 analog treatment caused a slight, but non-significant increase in cerebral glucose metabolism (CMR_glc_) after 6 months of treatment (275). As a decline of CMR_glc_ correlates with cognitive impairment, synaptic dysfunction and evolution of the disease, GLP-1's slight reduction as noted in this study offers a potential mechanism of benefit. However, a large gap in regards to AD and GLP-1 based therapies remains, since the small sample size of the study precluded the ability for the study to determine if GLP-1 administration reduces amyloid β (Aβ) load or alter cognitive scores (275). Nevertheless, in PD, an initial study into GLP-1 analog therapy, published in 2013, assigned 45 patients with moderate PD to receive subcutaneous Exenatide injections for 12 months alongside patient which did not receive any injection. Despite lacking a placebo-control in this study, the blinded ratings were indicative of clinical improvement in both motor and cognitive measures compared to control (277, 278). This study has since been expanded with a Swedish group assessing the effects of both GLP-1R agonists and DPP-4 inhibitors, and a UK based study opting for once weekly Exenatide treatments. The Swedish study, through use of a population-based nested case-control study, found a significantly decreased incidence of PD among individuals who had been recorded to take DPP-4 inhibitors (276). This is in contrast to the UK based studies (NCT01971242 and NCT01971242) which noted a positive and persistent effect of Exenatide treatment in off-medication motor scores (267).

Given the promising results from clinical trials, an understanding the mechanisms underlying GLP-1 mimetic actions on normal and diseased neural tissue would be invaluable. Neurodegenerative diseases share several pathological features, including but not limited to, synaptic loss and failure, reduced neurogenesis, enhanced free radical production, and cell death [reviewed in (279–282)]. The accumulation of misfolded proteins, common in neurodegeneration, impairs cellular communication and function, and causes the activation of neuronal inflammatory responses by activation of glial cells (microglia and astrocytes). Although such neuroinflammatory responses initially maintain homeostasis, chronic activation leads to increased severity of the disease state (283–285). Fortunately, GLP-1 effects in the brain are reminiscent of its actions on pancreatic β-cells, signaling through GLP-1R to initiate anti-inflammatory, anti-apoptotic and pro-survival effects (250, 286, 287).

The pro-survival effects of GLP-1 on neurons are attributable to the reduction of ER-stress and enhanced autophagy in neural cells. Studies by Panagaki et al. (288) as well as Chen et al. (289) reported that liraglutide enhanced AKT signaling and STAT3 activation, resulting in reduced apoptosis. These effects can also arise from activation of GLP-1R in astrocytes and microglia (290), which when triggered can reduce the levels of pro-inflammatory cytokines such as TNF-α and IL-1β in different models of brain inflammation (290–293). In PD, chronic activation of microglia can trigger polarization toward the cytotoxic M1 macrophages, leading to a self–perpetuating persistent inflammatory environment (294, 295), considered to be a major factor in driving dopaminergic degeneration (267, 296, 297). Use of Exenetide has been reported to halt dopaminergic degeneration and restore dopamine (DA) imbalance induced by 6-OHDA, MPTP, and Lipopolysaccharide in animal toxin models (271, 287, 298). Although mechanism of action through which GLP-1 stimulates microglial function in regards to chronic inflammation remain unclear, several studies point to NF-κB activation achieved through DPP-IV inhibitors in rotenone induced rodent PD models. Increased levels of NF-κB are observed in tyrosine hydroxylase (TH) and dopaminergic neurons, astrocytes and microglia, with implications on the pathogenesis of PD; while inhibition of NF-κB is correlated with neuroprotective effects in such PD models (299, 300). Since GLP-1 signaling activates AKT, one plausible explanation for its therapeutic effect in PD to reduce glial inflammation is through this increased activity in order to elevate levels of the Inhibitor of NF-κB (IκBα), ultimately leading to a reduction in neuroinflammation (26, 301).

Extending from these findings, GLP-1 mimetics can also act upstream of chronic neuronal inflammatory responses in cells by ameliorating the accumulation of misfolded proteins. The accumulation of misfolded proteins can occur through dysregulation of key cellular process, which in turn adversely affects neuronal homeostasis (267, 302–305). GSK-3 α/β isoforms are an example of constitutively active key regulatory enzymes, which when recruited and activated, by α-Synuclein (α-Syn) (a key mediator of PD), leads to hyperphosphorylation of Tau and subsequent increased accumulation of amyloid aggregates (306–309). Several *in vivo* and *in vitro* studies have showed that GLP-1 administration can protect neurons from Aβ aggregation, advanced glycation end products (AGEs) insult, and reduce tau hyperphosphorylation through regulation of GSK-3β signaling. The proposed mechanism of action of GLP-1 is believed to occur through activation the PI3K/AKT signaling pathway, leading to phosphorylation and deactivation of GSK-3β amino-terminal serine residue (271, 302, 310, 311). As previously indicated, GLP-1R activation may reduce protein aggregation through autophagic clearance, however a study by Yuan et al. (312) in 2015, indicated that rotenone induced alterations to autophagy and α-Syn clearance, are mediated by Ca^2+^/AKT/GSK-3β signaling pathway. Particularly, the authors reported that rotenone treatment of PC12 cells (derived from a tumor of the rat adrenal medulla) increased intra-cellular Ca^2+^ which, in turn, induce aggregation and phosphorylation of α-Syn and impair autophagy. While the hypothesis that raised intracellular Ca^2+^ promotes aggregation α-Syn is supported by previous work (313), the claim that rotenone induced alterations to autophagy and α-Syn, are mediated by Ca^2+^/GSK-3β signaling pathway may be an oversimplified statement, but nonetheless, reveals an additional mechanism by which GLP-1R signaling can alleviate protein aggregation. As Ca^2+^ is a second messenger in the cell, inappropriate fluctuations would undoubtedly impact autophagy and protein aggregation, and through the use of rotenone, an inhibitor of mitochondrial complex I, leads to elevation of intracellular Ca^2+^ through inhibition of resting background K^+^ currents, membrane depolarization, and VDCC opening, This would in turn impact ATP production, inhibit AC activity, down regulate cAMP signaling, and disrupt mitochondrial membrane potential (314, 315). Changes in cellular ATP, and mitochondrial stability, induced by protein aggregation would not only promote apoptosis, but increase cellular ROS, and oxidative stress, all of which act together to contribute to the destabilization of ER homeostasis and autophagy in neurodegenerative diseases (314, 316–319). GLP-1R activation can act to mitigate the deleterious effects of overloaded intracellular Ca^2+^, as mentioned before, through the cAMP/PKA/EPAC pathways and is thought to be an integral mechanism in the prevention of spatial memory and hippocampal synaptic plasticity impairments arising from Aβ-induced toxicity (43, 320, 321). The GLP-1 mediated regulation of Ca^2+^ is also coupled to restoration of insulin signaling throughout the brain, which can further promote its pro-survival abilities. This is crucial as impaired insulin signaling in AD and PD patients has been reported to negatively impact dendritic sprouting, neuronal stem cell growth and tissue repair (322–324).

Within the brain, endogenous, brain derived GLP-1 can promote insulin release (325, 326), thereby potentially increasing the expression of the internalized IR and IGF-1R in AD patients. Such a mechanism has recently been reported to restore IR signaling deficits through decreases of the c-Jun N-terminal kinase (JNK) signaling in symptomatic (?) T2D mice (326). These studies are furthered in rodent models whereby both chronic and acute treatments of liraglutide have been shown to protect against Aβ-induced impairment of memory and spatial learning in rats (315), as well as prevent memory impairment, reduce β-amyloid plaque, and plaque induced chronic inflammation in the APP/PS1 AD mice model (315, 327). AD rodent models chronically treated with the GLP-1 analog Val(8)glucagon-like peptide-1 caused modulation of neurotransmitter release, synaptic transmission (LTP) formation, and restoration of synaptic plasticity, as well as preventing impairment in the learning of new spatial tasks (328–330). Restoration of IR signaling, acts synergistically with GLP-1 signaling, modulating autophagy, oxidative stress, protein synthesis, apoptosis, and mitochondrial biogenesis (331, 332). GLP-1's oxidative stress protective mechanisms are indicated to be ameliorated in primary cortical neurons by GLP-1/CREB signaling inducing expression of apurinic/apryimidinic endonuclease 1 (APE1), a key enzyme of the base excision DNA repair (BER) pathway (333), while also impacting protein aggregation induced neurotoxicity through enhanced mitochondrial function. This has been indicated to occur through the deacetylase SIRT1 (334), its promotion of heat shock protein 70 (HSP70), an augmenter of normal α-Sync folding (335), modulation of PGC-1α, as well as activation of ADAM10, through retinoic acid receptor β, leading to the reduction of plaque formation (336).

It is evident that the mechanism through which GLP-1 stimulates pro-survival signaling, reduces neural tissue inflammation and improves cognitive function is complex. However, by defining cell-type specific signaling pathways in neural cells, it may be possible to develop distinct treatment strategies that uniquely modulate GLP-1 signaling in mental illness as well as neurodegenerative diseases.

## Conclusions

GLP-1 promotes glycemic control through a plethora of widely recognized physiological mechanisms. Among them, stimulation of insulin secretion and inhibition of glucagon release directly improve postprandial glucose homeostasis, while inhibition of gastric emptying and food intake represent a longer term positive effect of limiting weight gain. Due to these properties, GLP-1 therapies have been routinely and successfully used for the treatment of T2D and obesity for more than a decade. Most recent studies unveiled that GLP-1 analogs also act in the CNS and various peripheral tissues to restore and maintain normal cellular functions. This has been demonstrated in response to a variety of distinct disease paradigms and physiological insults, either through direct cell autonomous effects or through indirect whole body metabolic improvements. In this review, we provided a thorough description of the diverse roles for GLP-1R signaling across multiple tissues, focusing in the downstream pathways stimulated by acute and chronic activation of the receptor, and discussed novel pleiotropic applications of GLP-1 mimetics in the treatment of human disease. Continuing efforts to delineate tissue specific mechanisms of GLP-1 action are necessary in order to identify novel translational alternatives and foster the development of new GLP-1-based therapeutic agents harnessing different aspects of GLP-1 biology with therapeutic potential not only for T2D and obesity, but also for heart, liver, kidney, lung and brain related disorders.

## Author contributions

JR and RC conceived the idea, organized, and designed the manuscript. JR wrote the first draft, which was reviewed by RC, JH, and PN. Figures were designed by JR and RC. All authors contributed in manuscript revision and agreed with the final submitted version.

### Conflict of interest statement

The authors declare that the research was conducted in the absence of any commercial or financial relationships that could be construed as a potential conflict of interest.
